# Nitric Oxide-Producing Cardiovascular Stent Coatings for Prevention of Thrombosis and Restenosis

**DOI:** 10.3389/fbioe.2020.00578

**Published:** 2020-06-24

**Authors:** Jingdong Rao, Ho Pan Bei, Yuhe Yang, Yu Liu, Haodong Lin, Xin Zhao

**Affiliations:** ^1^Department of Biomedical Engineering, The Hong Kong Polytechnic University, Hong Kong, China; ^2^State Key Laboratory of Molecular Engineering of Polymers, Department of Orthopedic Surgery, Fudan University, Shanghai, China; ^3^General Hospital, Shanghai Jiaotong University School of Medicine, Shanghai, China

**Keywords:** cardiovascular stents, surface coating, nitric oxide, restenosis, thrombosis

## Abstract

Cardiovascular stenting is an effective method for treating cardiovascular diseases (CVDs), yet thrombosis and restenosis are the two major clinical complications that often lead to device failure. Nitric oxide (NO) has been proposed as a promising small molecule in improving the clinical performance of cardiovascular stents thanks to its anti-thrombosis and anti-restenosis ability, but its short half-life limits the full use of NO. To produce NO at lesion site with sufficient amount, NO-producing coatings (including NO-releasing and NO-generating coatings) are fashioned. Its releasing strategy is achieved by introducing exogenous NO storage materials like NO donors, while the generating strategy utilizes the *in vivo* substances such as *S*-nitrosothiols (RSNOs) to generate NO flux. NO-producing stents are particularly promising in future clinical use due to their ability to store NO resources or to generate large NO flux in a controlled and efficient manner. In this review, we first introduce NO-releasing and -generating coatings for prevention of thrombosis and restenosis. We then discuss the advantages and drawbacks on releasing and generating aspects, where possible further developments are suggested.

## Introduction

Cardiovascular diseases (CVDs) are a common cause of morbidity worldwide, accounting for 18 million deaths per year, and serve as a third of all global deaths (Frieden and Jaffe, 2018; Yusuf et al., 2020). To treat CVDs, surgical interventions including heart valve replacements (Saito et al., 2003), angioplasty (Stone et al., 2002), and intravascular stents (Zhu et al., 2014) are employed. Among these surgical procedures, cardiovascular stent is most commonly used in coronary heart disease, myocardial infarction, and stenocardia due to its effectiveness at dilating blood vessels and maintaining the circulation of blood (Kubo et al., 2008; Geng et al., 2013; Wei et al., 2013). In the United States alone, ~2 million patients undergo stent implantation each year (Cicha et al., 2016). However, stenting has a high tendency to cause thrombosis and restenosis. Intravascular injuries during surgical implantation stimulate coagulation pathways to facilitate platelet adhesion (Furie and Furie, 2008), and the production of thrombin further contributes to platelet activation and converses fibrinogen into fibrin-formed thrombus (Zhao et al., 2014). The accumulation of platelets and leucocytes causes acute inflammatory response and results in endothelial malfunction, which can lead to excessive proliferation of smooth muscle cells (SMCs) (Naghavi et al., 2013). The extracellular matrix (ECM) produced by SMCs then induces thickening of the vessel wall, contributing to neointimal hyperplasia and restenosis (Scott and Panitch, 2014). As a result, thrombosis and restenosis hamper the long-term effectiveness of cardiovascular devices and induce other related symptoms.

To ease the risk of thrombosis and restenosis, cardiovascular stents are modified with different polymers, biomolecules, or coatings. Compared to the traditional bare metal stents (BMSs) with unacceptable levels of thrombosis and restenosis (Nakamura et al., 2016), drug-eluting stents (DESs) can reduce neointimal hyperplasia and preserve vessel patency by releasing drugs from surface polymers (Joner et al., 2006). However, DESs may induce late thrombosis, which can be attributed to the depletion of drug reservoir, off-target effect of drugs, and the inflammatory response (Mcfadden et al., 2004; Ma et al., 2010). The most recent DES technology fabricates dual-therapy stents (DTSs) that combine two therapeutic regents like aspirin and adenosine diphosphate-receptor blockade for anti-platelet therapy (Ohkubo et al., 2011). Meanwhile, bioresorbable vascular scaffolds (BVSs) can decrease the propensity for thrombosis, since BVSs allow implants to degrade over time and leave an intact vessel (Stone, 2016). Furthermore, bio-engineered stents (BESs) adopt biocompatible materials, cell capture technology, or autologous venous tissue for better therapeutic effects (Stone et al., 2002; Hara et al., 2006; Colombo et al., 2019). In addition to material innovation, novel stent designs also employ coatings to improve the surface properties and clinical behaviors (Yang et al., 2020a). The coating materials possess the feasibility to directly attach or deposit onto the stents' surface and the ability to maintain a high local therapeutic concentration at specific site (Yang et al., 2017). Drugs and biomolecules such as paclitaxel (Palmerini et al., 2015), adhesive peptides (Wei et al., 2013), anti-CD 34 antibodies (Yoon et al., 2002), and nitric oxide (NO)-producing moieties (Gunasekera et al., 2018) are employed to improve the clinical behaviors of stents (see Table 1 for details). Although these molecules achieved good therapeutic effects, they still present some limitations. For example, high toxicity and non-selective function of paclitaxel raises safety concerns about its side effects while high cost and potential complications of antibodies and adhesive peptides may hinder treatment efficacy (Pacelli et al., 2017). Among all therapeutic molecules, while NO still possesses some drawbacks such as low diffusion distance in blood, it remains highly effective because of its anti-thrombosis and anti-restenosis ability. As an endogenous substance, its pro-proliferative activity to endothelium and anti-adhesion/aggregation effect on platelets additionally allow NO to serve as a guardian of cardiovascular implants (Winther et al., 2018). As reported by the ClinicalTrials.gov website, there are 678 NO-related clinical studies in CVD treatment by April 2020, which reveals the highly promising research and clinical application prospects of NO.

**Table 1:** Typical drugs and biomolecules for anti-thrombosis and anti-restenosis treatment.

**Type**	**Therapeutic substance**	**Mechanism of action**	**Therapeutic ability**	**Drawbacks**	**References**
Drugs	Paclitaxel	Inhibit cell proliferation, adhesion, and migration	Reduce intimal hyperplasia	Indiscriminate cell suppression, delayed re-endothelialization and impaired endothelial functions	(Lundberg et al., 2015; Palmerini et al., 2015)
	Sirolimus/zotarolimus	Interfere cell cycle by inhibiting the mammalian target of rapamycin	Promote re-endothelialization	Long-term safety concerns, late stent thrombosis	(Joner et al., 2006; Park et al., 2010)
Biomolecules	NO and NO-producing materials	Activate soluble guanylate cyclase related signal pathway	Vasodilatation, stimulate EC growth, inhibit SMC proliferation and platelet activation/aggregation	Short-lived, limited diffusion distance, by-products during NO-producing process	De (Mel et al., 2011; Carpenter and Schoenfisch, 2012)
	Antibodies (e.g., CD34 antibody)	Selectively recruit endothelial progenitor cells	Enhance growth of neointimal layer and prevent adhesion of thrombotic tissues	Complex preparation process, high cost, immune response	(Hristov et al., 2003; De Visscher et al., 2012)
	Cell-adhesive peptides (e.g., Arg-Glu-Asp-Val)	Mediate the adsorption and migration of targeted cells	Enhance re-endothelialization and anti-restenosis	Immune response, concerns on complications or undesired tissue growth	(Wei et al., 2013; Mahara et al., 2015)
	Growth factors (e.g., endothelial growth factor)	Promotes EC recruitment, adhesion, migration, and proliferation	Facilitate re-endothelialization	Short half-life, high dose with high cost	(Poh et al., 2010; Shin et al., 2012)
	Heparin	Inhibit the activation of thrombin	Possess anti-coagulant effect and reduce platelet aggregation	Hemorrhage and thrombocytopenia	(Gurbel and Bliden, 2003; Chuang and Masters, 2009)

In a healthy vasculature, endothelial cells (ECs) produce NO to achieve thrombotic homeostasis by preventing platelet activation (Seabra et al., 2015). The mechanism of NO inhibition of platelet activity is multifaceted. The main way is to activate the soluble guanylate cyclase (sGC), thereby increasing concentrations of cyclic guanosine monophosphate (cGMP) and stimulating the downstream platelet inhibition molecule—Protein Kinase G (PKG) (Francis et al., 2010; Kraehling and Sessa, 2017). The other anti-platelet activity of NO is to inhibit the thromboxane receptor on platelet membranes (Fuentes and Palomo, 2016). Meanwhile, NO is also closely related to cardiovascular homeostasis. In the cardiovascular system, NO can relax the surrounding smooth muscle, lead to vasodilation, and increase blood flow (Jon O Lundberg et al., 2015). Moreover, NO stimulates EC migration and suppresses SMC proliferation (Mel et al., 2011). It also influences angiogenesis and vascular remodeling as well as kills various pathogens (Devine et al., 2019).

**Figure 1:**
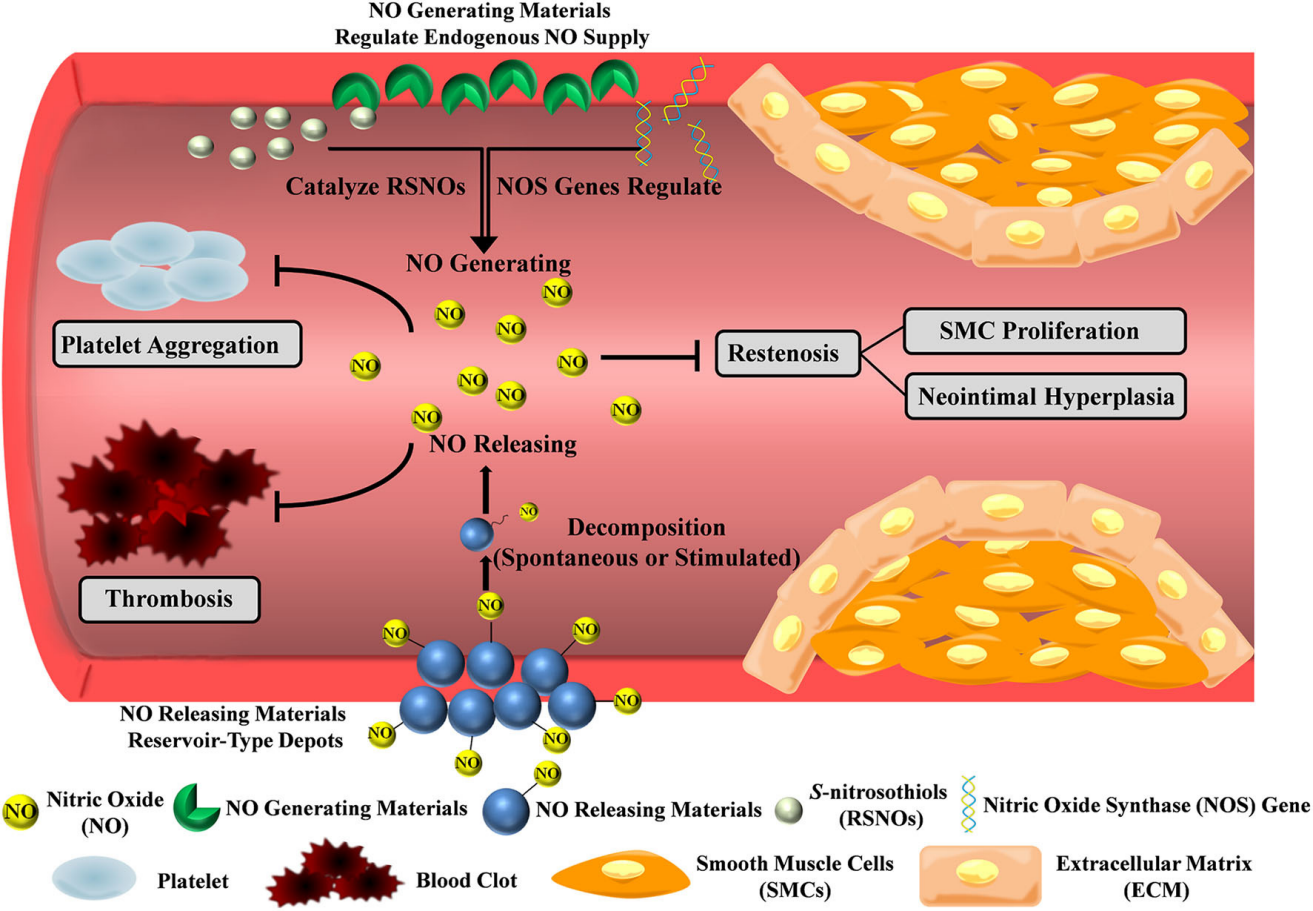
NO-producing strategies for prevention of thrombosis and restenosis. NO-releasing materials such as NO donors and peptides work as a finite reservoir, while NO-generating materials utilize catalytic substances or genes to achieve endogenous NO supply. The NO flux inhibits platelet activation and SMC proliferation, thereby preventing thrombosis and restenosis.

Under physical condition, NO can only diffuse around 100 μm and degrades within seconds in blood, so NO must be administered or produced at the diseased site (Thomas, 2015). Hence, incorporating NO production moieties with stent coatings will work as a functional platform to achieve the anti-thrombosis and anti-restenosis purpose. In this review, we will discuss NO-producing coatings, with the emphasis on the NO-releasing and -generating strategies (Figure 1 and Table 2). NO-releasing materials such as NO donors or peptides work as a finite reservoir, while NO-generating strategies utilize catalytic substances or genes to achieve endogenous NO supply. These coatings overcome the challenges of NO administration, and serve as great delivery platforms since they can maintain the physiologically relevant concentrations of NO at a specific site.

**Table 2:** NO-producing strategies in anti-thrombosis and anti-restenosis treatment.

**Category**	**Functional substance**	**Mechanism of NO production**	**Advantages**	**Drawbacks**
NO-releasing materials	NO donors (e.g., NONOates, SNAP)	Release NO at physiological condition by heat, light, copper ions	Store NO molecules	Limited releasing period, instability of the NO donors, finite reservoir of NO
	NO prodrugs (e.g., Gal-NONOates)	Release NO upon enzyme-triggering	Keep NO donors stable, prolong the releasing time	Drug waste and side effects by systemic delivery, finite reservoir of NO
	Peptide amphiphiles	Spontaneously release NO from NO donating residues	Mimic biochemical properties, prolong the releasing time	High cost, difficulties in peptide activity control, finite reservoir of NO
NO-generating materials	Gene therapy	Initiate NOS production	Achieve durable regulation by long-term expression	Instability, deliverability, release kinetics, off-target transduction, and high cost
	Catalytic approaches (e.g., Se, Cu^II^)	Catalyze decomposition of RSNOs by GPx-like catalytic substances	Exhibit significant long-lasting NO production with relative high NO flux	Lack of smart control in different disease stages
Combination strategy	Integration of NO release and generation (e.g., SNAP with copper nanoparticles, EPT with ZnO particles)	Ensure the supply of both exogenous and endogenous of NO	Achieve flexible, extended and controllable NO supply, be suitable in various therapeutic needs	Raise excessive NO production concerns

## No-Releasing Coatings

In NO-releasing strategies, the potential NO can be stored in small molecules or biomolecules, and through connecting, dispersing, or encapsulating those functional molecules as a part of delivery system, the platform will act as a reservoir for conditional and spontaneous release of NO.

### NO Donors

NO donors [e.g., N-diazeniumdiolates (NONOates), *S*-nitroso-*N*-acetylpenicillamine (SNAP)] are pharmacologically active substances that carry NO for localized release (Major et al., 2010; Midgley et al., 2019). NONOates can be synthesized by reacting gaseous NO with secondary amines under high pressure (5 atm) and spontaneously decomposed to release two moles of NO per mole of donor at physiological condition (Midgley et al., 2019), while SNAP, a kind of synthetic *S*-nitrosothiols (RSNOs), requires reactions between nitrosating agents and thiols, and NO can be exhausted from SNAP by heat, light (340 and 590 nm), or copper ions (Naghavi et al., 2013).

For example, Joung et al. fabricated NONOates containing liposome stent coatings via layer-by-layer (LBL) method to control the release of NO (Elnaggar et al., 2016). The NO release profiles showed that the release rate sustained up to 5 days with good thrombo-resistant effect. As thrombus involves the formation of fibrin and activation of platelets, Yang et al. proposed a novel concept to integrate anti-coagulant agent bivalirudin (BVLD) and NO as a bulk synergetic modification on surface coating (Yang et al., 2020b). BVLD was covalently connected with the primary amine groups on plasma polymerized allylamine (PPAm)-coated surface, and then the coating was immersed in basic solution under high-pressure NO gas to form a NONOate-like bulk depot. For circumventing NO burst release, hydrophobic hexafluoroethane was introduced into this system, which prolonged the release time of NO to more than 8 h. Compared to 316L SS, the occlusion rate and thrombus weight in BVLD/NO-PPAmF were reduced from 58.7 ± 3.9% to 3.3 ± 1.2% and 64.1 ± 3.9 mg to 1.7 ± 1.2 mg after 2 h of extracorporeal circulation (ECC), respectively. Since the complexity of thrombogenic process requires multi-therapy procedures, the combination of anti-platelet and anti-coagulant dual functions is a promising strategy to integrate into cardiovascular coatings.

However, the NO release period in these studies was limited. To extend the NO release time, Hopkins et al. designed a highly stable NO-releasing coating by covalently attaching SNAP with poly(dimethylsiloxane) (PDMS) (Hopkins et al., 2018). Previous studies demonstrated that SNAP-based polymers have exhibited significant leaching of SNAP, which will result in non-localized NO release (Brisbois et al., 2016). Through tethering to PDMS, SNAP leaching into surrounding environment was prevented and allowed for lengthened potency. The NO flux formation of SNAP-PDMS reached up to 125 days *in vitro* and maintained at 0.1 × 10^−10^ mol × cm^−2^ × min^−1^ till the end of the testing period. The SNAP-PDMS showed long-duration anti-bacterial efficacy, and it exhibited 78% thrombus reduction (compared with blank control) after testing by ECC over 4 h. Although this strategy achieved long-term NO release, the low NO levels in the later release stages limited the long-lasting anti-thrombotic effect.

Despite these efforts on NO donor applications, the long-term therapeutic effect after implantation was unsatisfying. Integrating NO-donor loaded liposomes with stents faces the risk of low loading capacity, carrier detachment from the stents and delayed NO release. Chemical covalent bond offers bulk NO donor storage, but it also brings extra reaction regents and shows relative low NO flux. Besides, the instability of the NO donors is a big obstacle that cannot be solved by simple connection or encapsulation. Hence, many other coatings have been developed for better application.

### NO Prodrugs

Since NO donors are unstable under thermal, acidic, or physiological conditions, researchers use enzyme prodrug therapy (EPT) to maintain the stability of donors and control the release (Chandrawati et al., 2017). By fabricating NO donors (e.g., NONOates) with enzyme-sensitive linkers, the formed prodrugs remain stable and inactive in non-enzymatic environment and only release NO upon enzyme-triggering.

In a study of NO EPT, Wang et al. designed a galactosidase (Gal) immobilized surface coating and prepared glycosylated NONOate (Gal-NONOate) as NO prodrug (Wang et al., 2015). After the *in vivo* implantation of enzyme-functional platform, Gal-NONOate was administrated by tail vein injection, it then circulated until contact with the coating and the enzyme would catalyze the decomposition of the prodrug to release NO locally. The *in situ* release of NO was verified using a fluorescent probe to trace the NO flux. The immobilized enzyme retained the catalytic property up to 1 month *in vivo* and the results revealed effective inhibition of thrombosis and enhancement of vascular tissue regeneration and remodeling in EPT group. To engineer EPT for diverse medicinal implants, Zelikin et al. optimized EPT coating by adopting LBL method to fabricate multilayered polyelectrolyte coating with immobilized β-Gal enzyme (Winther et al., 2018). This method was all-aqueous and solution-based, which could accommodate modification of any substrate with no restriction on surface topography and geometry.

In addition to maintaining NO donors' stability, through using different concentrations of prodrugs or regulating administration time to adjust physiological effect, EPT can achieve personalized, fine-tuned therapeutic delivery of NO (Pan et al., 2015). Nevertheless, EPT requires systemic delivery of NO prodrugs, which only has effect at the enzyme-modified lesion site. This means that even with a bulky administration, only a small portion of drugs would work, which causes drug waste and side effects due to the high cytotoxicity of the drug. Scientists thus have developed other biomolecules to achieve NO release.

### NO-Releasing Peptide Amphiphiles

Peptide amphiphiles (PAs) consist of hydrophobic tails coupled to hydrophilic functional peptide sequences that are attractive in biomimetic scaffolds, since enzyme-mediated degradable sites and cell adhesion ligands can be incorporated into PAs to mimic some biochemical properties (Jun et al., 2005; Cheng et al., 2015).

In a report by Matson et al., a NO-releasing PA coating was designed (Kushwaha et al., 2010). The functional peptide sequences involved a matrix metalloprotease-2 (MMP2) mediated cleavage site Gly-Thr-Ala-Gly-Leu-Ile-Gly-Gln (GTAGLIGQ), coupled to an EC-adhesive ligand Tyr-Ile-Gly-Ser-Arg (YIGSR) or a polylysine (KKKKK) group to form NO donating residue peptide. Burst release of NO occurred within 48 h, followed by sustained release for 30 days. This functional peptide increased initial adhesion of ECs from 51 ± 3% to 67 ± 2%, while the proliferation of SMCs were inhibited from 35 ± 2% to 16 ± 3% after 48 h of incubation. Additionally, compared with the positive control collagen group, the peptide group showed 150-fold decrease in platelet attachment, suggesting the potential of such coating for modification of various cardiovascular implants. Similarly, Alexander et al. used the same PAs to form a nanomatrix coating (Alexander et al., 2016), and they proved the vasodilatory effects *ex vivo* and the anti-inflammatory ability *in vitro*. This coating could address the shortcomings of the implanted stents and had the potential to be developed in animal models with cardiovascular stents. However, it is difficult to popularize peptide clinical application due to the high cost of peptides and the lack of control over their activity.

NO-releasing strategies have showed appropriate therapeutic effects on thrombosis and restenosis, but they still possess some shortcomings. In addition to the problems of different NO-releasing materials discussed above, all these strategies have to face the biggest obstacle—the finite reservoir of NO. With the NO release reaction proceeding, the elements will be exhausted within a short time frame. Therefore, reliable long-term and sufficient NO supplies need to be achieved.

## No-Generating Coatings

Radical NO species are short-lived. To overcome this drawback, NO-generating materials were developed, which utilize genes and catalytic substances such as selenium (Se) or copper ions to stimulate NO production. These strategies can achieve sufficient and long-term NO release depending on the endogenous regulation and continuous supply of NO resources.

### NOS Gene Therapy

In the cardiovascular system, nitric oxide synthase (NOS) especially endothelial NOS (eNOS) and inducible NOS (iNOS) play a very important role. eNOS is highly related to the function of the endothelium, which can catalyze the production of NO via enzymatic effect (Zhao et al., 2013). iNOS is normally absent in the vasculature under physiological conditions, but it expresses in blood vessels under pathological situations (Sessa, 2004), and it can generate large amounts of NO over long periods of time (Kleinert et al., 2005). Since NOS has emerged as an active player in NO generation, a lot of therapeutic strategies have been developed around it. Gene therapy in CVDs can improve the selectivity of exerted effects toward certain cells and has the potential for combination therapy. Many researchers have thus investigated the therapeutic effect of NOS genes (Forstermann et al., 2017). In a study, DiMuzio et al. developed a natural vascular tissue mimetic stent by seeding it with autologous adipose-derived stem cells (ASCs) differentiated endothelial-like cells, and they used the eNOS gene to transfect these ASCs (McIlhenny et al., 2013). The transfection initiated eNOS production, yielding eNOS to generate functional NO gas. They found that the transfected ASCs produced NO (247 ± 10 nM) at a similar level to EC controls (288 ± 29 nM) *in vitro*, and exhibited a non-thrombogenic surface compared to unseeded controls *in vivo*.

In addition to *in vitro* gene transfection therapy, gene delivery is another approach to achieve targeted protein expression. To deliver NOS gene, Levy et al. described a gene delivery platform that provided local arterial gene transfer via iNOS-encoding adeno-associated virus serotype 2 (AAV2) vectors (Fishbein et al., 2017). Through affinity effect, the iNOS-cDNA sequence loaded AAV2 vectors could be immobilized onto stent surfaces. Compared to the non-gene group, AAV2_iNOS_ showed a 16-fold higher NO production *in vitro*. AAV2_iNOS_ demonstrated escalating expression of encoded transgene for 12 weeks and showed the anti-restenosis efficacy with 95% inhibition rate *in vivo*.

Although there are many attempts in using NOS gene strategies, the instability, deliverability, release kinetics, off-target transduction, and high cost of gene limit the clinical application. Researchers are hence working hard on more effective NO-generating pathways.

### Endogenous NO Catalytic Approaches

RSNOs, a kind of endogenous NO donors in the blood, has highly promising opportunities to achieve localized synthesis of NO for continuous supply (Li et al., 2018). It was found that glutathione peroxidase (GPx) and Se or copper ions with GPx-like catalytic activity can catalyze the decomposition of RSNOs into NO *in vivo* (Weng et al., 2011; McCarthy et al., 2016).

**Figure 2:**
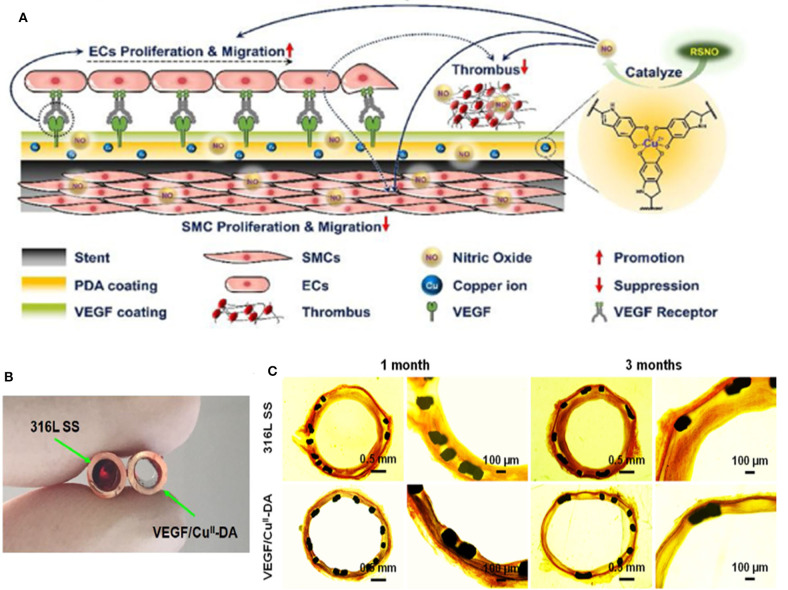
Cardiovascular stents with VEGF/Cu^II^-DA coatings for enhanced re-endothelialization and reduced thrombosis. **(A)** Mechanism of thrombosis and restenosis prevention of VEGF/Cu^II^-DA-coated stents. The spatiotemporal dual delivery of VEGF and NO enabled the fast re-endothelialization of stents and prevented thrombosis and restenosis. **(B)** Images of cross-sectioned tubing containing uncoated and VEGF/Cu^II^-DA-coated 316L SS foils exposed for 1.5 h to blood flow in a rabbit arteriovenous shunt model. **(C)** Histomorphometric analysis on restenosis at bare 316L SS and VEGF/Cu^II^-DA-coated stents using Van Gieson's staining. Image source: Tu et al. (2020).

Recently, our group developed one-pot approach to incorporate selenocystamine (SeCA) in the framework of dopamine (DA) via co-immobilization (Yang et al., 2018). The NO release rates could be controlled (from 0.5 to 2.2 × 10^−10^ mol × cm^−2^ × min^−1^) by regulating the SeCA–DA molar ratio. The study showed that the SeCA/DA coating could release NO for more than 60 days, which achieved long-lasting prevention of thrombosis and restenosis. Additionally, the simple operation avoided the tedious process and toxic chemicals. This coating is believed to be universally formed on diverse types of materials with great clinical potential. We further improved the NO production ability by employing Cu^II^ instead of SeCA, since Cu^II^ possesses superior NO catalytic efficacy (Zhang et al., 2019). The NO release rates could reach to natural endothelium rates (0.5 to 4 × 10^−10^ mol × cm^−2^ × min^−1^) by adjusting the dose of Cu^II^ with NO flux ranging from 0.4 to 6.5 × 10^−10^ mol × cm^−2^ × min^−1^. After implantation for 3 months, the DA-Cu^II^-coated stents not only inhibited thrombosis, but also promoted re-endothelialization and reduced neointimal formation. We then additionally endowed our stents with anti-bacterial properties (Tu et al., 2019). In this system, cystamine (CySA) was chosen to fabricate the metal-phenolic-amine-based coatings with gallic acid (GA) and Cu^II^. GA was added as chelating agent with anti-bacterial properties, which worked synergistically with Cu^II^ in bacterial inhabitation. The integrated coating showed 99% anti-bacterial rate *in vitro* and 3.4 ± 0.2% occlusion rate *in vivo* after implantation for 30 days. In our most recent study, we further immobilized vascular endothelial growth factor (VEGF) with DA-Cu^II^ coating (Figure 2A) (Tu et al., 2020). The introduction of VEGF was found to accelerate the early-stage EC adhesion, migration, and growth, forming a new and complete endothelium on the stents. The NO flux generated by the DA-Cu^II^ coatings successfully suppressed thrombosis (Figure 2B). Scanning electron microscope images showed that after 1 and 3 months of implantation, VEGF/Cu^II^-DA coating demonstrated greater re-endothelialization compared to 316L SS (Figure 2C). Altogether, the multiple programmed therapeutic strategies for spatiotemporal dual delivery of NO and VEGF significantly prevented thrombosis and restenosis.

Nevertheless, these RSNO catalytic stents face difficulty in smart control of NO production. Additionally, different stages of disease and therapeutic purposes require different amounts of NO, a flexible, extended, and controllable NO supply is therefore highly sought after.

## No-Releasing and -Generating Integrating Coatings

There are substantial achievements of the anti-thrombosis and anti-restenosis therapeutic effects of NO-releasing and -generating strategies, but limitations still exist. Researchers thus make effort to combine those two strategies to supplement NO delivery. Maintaining NO release within the physiological region (0.5 to 4 × 10^−10^ mol × cm^−2^ × min^−1^, and preferably at the up end) can be more efficient for biomedical applications, but the NO donors such as SNAP have been shown to release NO near the low end of the physiological levels. Based on this, Handa et al. incorporated SNAP in a medical grade polymer coated with copper nanoparticles to achieve better regulation of NO release (Pant et al., 2017). This strategy not only ensured the supply of exogenous NO by SNAP, but also provided the enzymatic NO release via the reaction of copper ions on endogenous RSNOs in the blood. The Cu-SNAP had increased NO flux values to around 4.48 or 4.84 × 10^−10^ mol × cm^−2^ × min^−1^ when using different dosage of Cu. Additionally, significant reduction in bacterial growth and effective prevention of platelet adhesion has been achieved. Similarly, Brisbois et al. developed a multi-layered SNAP-doped polymer with a blended Se interface, and they focused on the different needs during the therapy process (Mondal et al., 2019). The first few hours after device implantation are crucial in preventing infection, since biofilm formation can occur rapidly after insertion (<24 h). The initial NO flux of generating materials may, however, be inadequate in preventing platelet activation or infection at early onslaught. Hence, in this strategy, SNAP offered initial NO release and Se interface could continuously generate NO in the presence of RSNOs. The results showed that there was a burst NO release on day 1, then the coating maintained a high and continuous NO release in the subsequent days. As a result, the enhanced initial NO flux would provide a potent anti-microbial activity acutely at the time of surgical placement, while the continuous NO generation contributed to inhibiting blood clot formation and protecting chronic or late device infections.

In addition to the above strategies to regulate the NO flux by using different coating compositions, Chandrawati et al. incorporated zinc oxide (ZnO) particles with EPT (Yang et al., 2020c). ZnO possess innate glutathione peroxidase and glycosidase activities, which allowed ZnO to catalytically decompose both endogenous (RSNOs) and exogenous (β-Gal-NONOate) donors to produce NO at physiological conditions. This strategy could solve problems such as finite pool of NO donors, short shelf life and low stability of natural enzymes in EPT. ZnO preserved its catalytic property for at least 6 months and the activity in producing NO was demonstrated. By tuning ZnO and NO prodrugs, physiologically NO levels were achieved. This method will be beneficial in long-term NO production and extra NO supplement can be achieved by on-demand NO prodrug administration, which has promising development in diverse blood-contacting devices.

In a word, the combination of NO-releasing and NO-generating chemistries within a single platform allows for a release profile that achieves the best of both worlds. However, further studies should effectively control the amount of NO flux to avoid excessive NO production and related potential toxicity.

## Conclusion and Future Work

The progress of the NO-producing stent coatings are highly inspiring in the prevention of thrombosis and restenosis, and researchers have also attempted to improve the fabrication process and committed to developing simple materials for future clinical application. However, the immune reaction, inflammation, anaphylaxis, and biocompatibility of the coatings need to be further investigated. Although these problems cannot be solved temporarily, in the long term, what can be done is to promote the development of NO production strategies from those achievable directions: (1) Combination strategies of NO and other pathway regulation need to be carefully considered about the dosage ratio between the drugs to avoid the antagonism effect and ensure synergistic effect. (2) The toxicity and metabolism of the NO reaction, by-products [like N-Acetyl-D-penicillamine (NAP) and disulfide] should be taken into account. (3) Scientists should develop precise methods of NO quantitative detection *in vivo* to meet different needs. (4) Smart NO production system can be further developed to respond to the microenvironment changes and to produce NO flux on-demand during different pathologic process. (5) Most researchers use healthy animals to evaluate the effect of NO; however, differences between the healthy and the CVD individuals should be assessed. Researchers should also combine the effects of NO-producing coatings and the implanted materials as a whole to investigate the therapeutic effect in their corresponding disease models, which will have much more clinical significance.

In summary, NO-releasing and NO-generating strategies still have significant room for improvement, and a thorough understanding of NO production and solutions for the corresponding pathological reaction will ensure the safe clinical practices for the next generation of technological devices. We hope that this review can be helpful for the further development in this research field.

## Author Contributions

XZ and HL supervised the whole review. JR and HP wrote the manuscript. YY and YL performed literature search and revised the manuscript. All the authors approved the review for publication.

## Conflict of Interest

The authors declare that the research was conducted in the absence of any commercial or financial relationships that could be construed as a potential conflict of interest.
